# The Impact of Targeted Therapy on Intracranial Metastatic Disease Incidence and Survival

**DOI:** 10.3389/fonc.2019.00797

**Published:** 2019-08-23

**Authors:** Anders W. Erickson, Sunit Das

**Affiliations:** ^1^Institute of Medical Science, Faculty of Medicine, University of Toronto, Toronto, ON, Canada; ^2^Division of Neurosurgery, University of Toronto, Toronto, ON, Canada; ^3^Li Ka Shing Knowledge Institute, St. Michael's Hospital, Toronto, ON, Canada

**Keywords:** intracranial metastatic disease (IMD), brain metastases, targeted therapy, survival, incidence

## Abstract

Intracranial metastatic disease (IMD) is a common and severe complication of primary cancers. Current treatment options for IMD include surgical resection and radiation therapy, although there has been recent interest in targeted therapy in the management of IMD. As of yet, insufficient data exist to support the recommendation of targeted therapies in the treatment of IMD. Paradoxically, targeted therapy has been hypothesized to play a role in the development of IMD in patients with primary cancers. This is based on the observations that patients who receive targeted therapy for primary cancer experience prolonged survival, and that prolonged survival has been associated with increased incidence of IMD. Few data exist to clarify if treatment of primary cancers with targeted therapies influences IMD incidence. Here, we discuss the role of targeted therapy in IMD management, review the current literature on IMD incidence and targeted therapy use in primary cancer, and propose the need for future studies to inform physicians in choosing treatment options and counseling patients.

## Introduction

The development of intracranial metastatic disease (IMD) complicates the course of approximately 20% of patients with cancer, with the highest frequency of brain metastases arising in patients with melanoma (7–16%), breast cancer (5–20%), and lung cancer (20–56%) (1–3). The consequences of IMD are severe: across all cancers, patients with IMD have a 2-year survival of 8.1% (1). Prognosis is informed by patient age, Karnofksy performance status, extent of disease, and in recent years, molecular marker status, such as HER2/neu in breast cancer and EGFR in non-small cell lung cancer (4). Importantly, molecular marker status has also opened up the possibility for treatment of brain metastases with targeted therapies.

Targeted therapies are medications that inhibit cancer-specific driver mutations. For example, vemurafenib is a small molecule inhibitor of the B-raf/MEK pathway specific for cells possessing the V600E BRAF mutation. The B-raf/MEK pathway is a driver of cancer cell proliferation and survival in BRAF-mutant melanoma; inhibition of this pathway with vemurafenib results in programmed cell death in these melanoma cells (5). The arrival of targeted therapies has revolutionized cancer treatment and improved outcomes for many patients with cancer. However, little is known about role of targeted therapies in the treatment of patients with IMD, or if targeted therapies modify the risk of development of IMD in patients with systemic cancer. Some targeted therapies have been shown to improve survival in patients with brain metastases, a cohort deemed previously to harbor a uniformly poor survival (1).

### Targeted Therapies and Survivorship in IMD

The therapeutic options that have historically been considered for treatment of IMD include surgical resection and radiation therapy; chemotherapies have not generally been useful in the treatment of brain metastases (6). Surgical resection has historically been reserved for patients with good Karnofsky performance status (KPS >70), well-controlled systemic disease, and a single or few accessible tumors (1, 7). Stereotactic radiosurgery (SRS), a therapy previously recommended for treating patients with up to four brain metastases (or >4 with cumulative volume <7 mL), is broadening its scope, and is now in clinical trial for patients with up to 20 brain metastases (NCT03075072) (8). Whole brain radiation therapy (WBRT) has historically been used as frontline therapy in patients with multiple brain metastases, but has been associated with neurocognitive decline in areas of episodic memory, executive function, processing speed, and fine motor control (6, 9, 10). Neuroprotective strategies adjunct to WBRT, such as memantine administration and hippocampal sparing, have been shown to reduce some of the deleterious neurocognitive effects of WBRT (6, 9, 10). Interest therefore exists in augmenting the treatment landscape, and replacing or delaying upfront radiotherapy with another treatment modality, such as targeted therapies (11).

Unfortunately, the data available in the literature on survival in patients with IMD treated with targeted therapies are limited and mixed. Existing studies support the hypothesis that patients who receive targeted therapies for the treatment of IMD experience prolonged survival (11–15). However, these studies have been limited by including only single study arms or too few patients, and have largely restricted their focus to IMD arising from single primary cancer subtypes. Some contradictory data also exist suggesting decreased overall survival (OS) with the use of targeted therapy for patients with IMD (16). Additionally, new-generation targeted therapies, such as alectinib and osimertinib, have been approved in only the last few years, and little is known about their outcomes on a population scale, although trial data suggest CNS efficacy (17, 18). At this time, the 2019 guidelines from the Congress of Neurological Surgeons cite insufficient evidence to recommend targeted therapies in treating IMD (19).

### Targeted Therapies and IMD Incidence

One factor of import in addition to considering the effect of targeted therapies on survival in patients with IMD is the effect of these drugs on patient survival independent of the development of IMD. Targeted therapies have been shown to improve systemic disease control and prolong OS in patients with multiple cancer subtypes (20–23). Some literature supports the hypothesis that prolonged survival in patients with cancer is associated with increased incidence of IMD (3, 24, 25). In other words, targeted therapies for primary cancer may paradoxically be associated with increased incidence of brain metastases by extending patient survival through improved control of systemic disease, while relegating the brain as a “sanctuary” site in which undetected intracranial micrometastases are sheltered from systemic treatment that is unable to penetrate the “sanctuary” of the blood-brain barrier (BBB) (14, 24–29). For example, a meta-analysis of three randomized trials found that patients taking trastuzumab for HER2/neu-positive breast cancer had improved OS, but were 1.82 times more likely to develop IMD than non-trastuzumab comparators (29). Similarly, in patients with BRAF-mutant melanoma, one retrospective study found that 90 patients taking BRAF inhibitors were 30% more likely than a chemotherapy comparator group to develop IMD, although these results were not significant (*p* = 0.5129), nor did the study report data comparing OS in patients without IMD (14). In patients with EGFR-mutant non-small cell lung cancer, patients receiving first-line EGFR-targeted therapies had improved OS, but were 1.35 times more likely to develop IMD compared with patients receiving other therapies (28), although other analyses suggest the same first-line EGFR-targeted therapies decrease the incidence of IMD (30, 31).

Conversely, some have postulated that newer targeted therapies that are capable of crossing the BBB may *decrease* the incidence of IMD by overcoming the sanctuary effect. A randomized controlled trial of alectinib (BBB-penetrant) vs. crizotinib (less BBB-penetrant) for ALK-positive non-small cell lung cancer showed 12-month cumulative incidences of central nervous system progression of 9.4 and 41.4%, respectively (18, 32). Importantly, alectinib did not offer these patients a survival benefit beyond that gained by therapy with crizotinib: the 12-month survival rate was 84.3% (95% CI 78.4–90.2) for patients receiving alectinib, and 82.5% (95% CI 76.1–88.9) for patients receiving crizotinib. In contrast, targeted therapies for renal cell carcinoma (RCC) have been reported to decrease incidence of IMD compared to chemotherapy, despite minimal BBB penetration of these therapies due to active efflux by transporters P-glycoprotein and breast cancer resistance protein (33).

**Figure 1:**
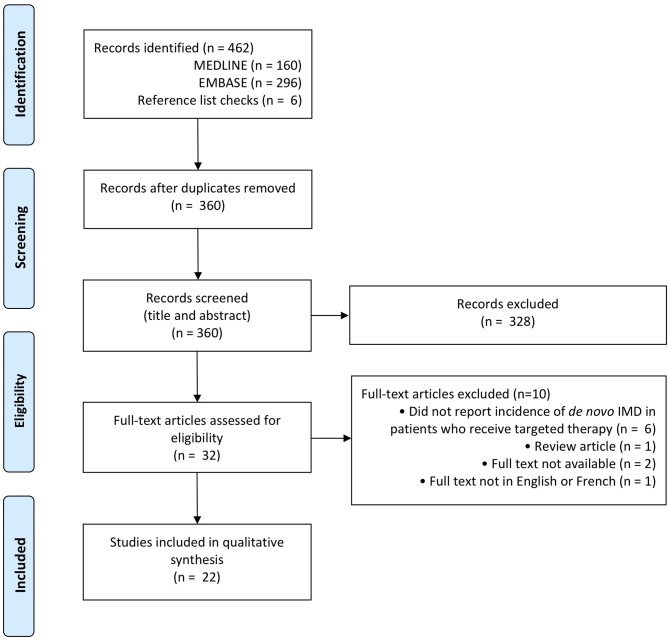
PRISMA flow diagram for IMD incidence with targeted therapy (50).

**Figure 2:**
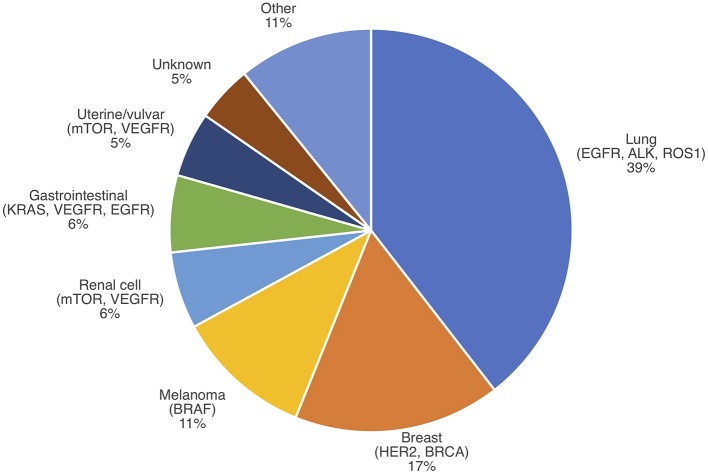
IMD incidence by primary cancer type with select actionable mutations. Modified from Nussbaum et al. (51).

A snapshot of the current literature reveals that knowledge of the impact of targeted therapy on IMD incidence is sparse (Table 1, Figure 1, Appendix 1 in Supplementary Material). Few studies address the question of IMD incidence following targeted therapy in comparison to the volume of literature on IMD *survival* with targeted therapy. Notably, there appear to be more studies on IMD incidence from breast cancer and non-small cell lung cancer in comparison to melanoma, RCC, and hepatocellular carcinoma (HCC). This may be because targeted therapies for breast cancer and non-small cell lung cancer have existed longer, and in greater number, than for melanoma, RCC, and HCC. This is also consistent with the observed distribution of primary cancers that contribute to IMD prevalence, which attributes 56% of IMD cases to lung and breast cancers (Figure 2) (52). Regardless of primary disease type, most of the literature is comprised of retrospective cohort studies at single institutions, limited to several hundred patients, or lacking controls. Some studies are prospective or meta-analyses, but these form the minority.

**Table 1:** Select studies reporting on IMD incidence in patients receiving targeted therapy.

**Disease**	**References**	**Therapy**	**Study type**	**Patients (*n*)**	**IMD incidence with targeted therapy**	**Findings**
Breast Cancer	Berghoff et al. (34)	Trastuzumab, lapatinib	Retrospective cohort	201	—	IMD incidence trended toward lower in trastuzumab (38.2%) vs. no trastuzumab (57.1%, *p* = 0.058). IMD incidence trended toward lower in lapatinib (30.8%) vs. no lapatinib (39.6%, *p* = 0.530).
	Swain et al. (35)	Pertuzumab vs. placebo (each with trastuzumab + docetaxel)	RCT	808	—	IMD incidence trended toward higher in pertuzumab arm (13.7%) vs. placebo arm (12.6%). But, median time-to-CNS-metastasis greater in pertuzumab arm (15.0 months) vs. placebo arm (12.9 months; HR, 0.58; 95% CI 0.39–0.85; *p* = 0.0049).
	Viani et al. (29)^*^	Trastuzumab vs. no trastuzumab	Meta-analysis	6,738	Higher	IMD incidence higher in trastuzumab arms by 1.82-fold (95% CI 1.89–3.16; *p* = 0.009).
	Bria et al. (36)^*^	Trastuzumab vs. no trastuzumab	Meta-analysis	6,738	Higher	IMD incidence higher in trastuzumab arms (RR, 1.57; 95% CI 1.03–2.37; *p* = 0.033).
	Okines et al. (37)	Ado-trastuzumab emtansine	Retrospective cohort	39	—	IMD incidence 18% in patients receiving ado-trastuzumab emtansine, with median time-to-IMD 7.5 months (95% CI 3.8–9.6). No control.
	Musolino et al. (38)	Trastuzumab vs. no trastuzumab	Retrospective cohort	1,429	Higher	IMD incidence higher in patients receiving trastuzumab (10.5%) vs. no trastuzumab (2.9%). HER2+ status and trastuzumab, together, predictive for CNS events (HR, 4.3; 95% CI 1.5–11.8; *p* = 0.005).
	Yau et al. (39)	Trastuzumab	Retrospective cohort	87	—	IMD risk not observed to be higher than disease-free population (RR, 1.0; 95% CI 0.4–2.2; *p* = 0.09). No control.
Melanoma	Sloot et al. (14)	BRAF/MEK inhibitor vs. chemo	Retrospective cohort	610	—	IMD incidence not higher in BRAF inhibitor vs. chemotherapy (OR, 1.3; 95% CI 0.6–2.49; *p* = 0.5129).
	Peuvrel et al. (40)	Vemurafenib	Retrospective cohort	86	—	IMD incidence 20% in patients receiving vemurafenib, with median time-to-IMD 5.3 months (±4.3). No control.
NSCLC	Heon et al. (31)	EGFR inhibitor	Retrospective cohort	81	Lower	IMD incidence lower in EGFR inhibitor arms (25% at 42 months) vs. historical comparators (40–55% at 35–37 months). No study control.
	Wang et al. (28)	EGFR inhibitor vs. other therapy	Retrospective cohort	1,254	Higher	IMD incidence higher in EGFR inhibitor vs. other therapy (HR,1.36; 95% CI 1.14–1.64; *p* = 0.001).
	Su et al. (41)	Gefitinib vs.Erlotinib vs.afatinib	Retrospective cohort	219	—	IMD incidences at 24 months for gefitinib (13.9%), erlotinib (9.3%), and afatinib (28.3%) were not significantly different (*p* = 0.80). Hazard ratio for IMD in afatinib vs. gefitinib 0.49 (95% CI 0.34–0.71; *p* = 0.001)
	Fu et al. (42)	Bevacizumab + chemo vs. chemo	Retrospective cohort	159	Lower	IMD incidence at 24 months lower in the bevacizumab + chemo arm (14.0%) vs. chemo arm (31%, p <0.01).
	Ilhan-Mutlu et al. (43)	Bevacizumab vs. chemo	Retrospective cohort	1,043	Lower	IMD incidence at 24 months lower for bevacizumab (2.6%) vs. chemo (5.8%, *p* = 0.01; HR, 0.36; 95% CI 0.19–0.68; *p* = 0.001).
	Gadgeel et al. (18)	Crizotinib vs. alectinib	RCT	181	—	IMD incidence at 12 months lower for alectinib (4.6%; 95% CI 1.5–10.6%) vs. crizotinib (31.5%; 95% CI 22.1–41.3%). Time-to-CNS progression longer in alectinib vs. crizotinib (csHR, 0.14; 95% CI 0.06–0.33; *p* < 0.0001).
	Nishio et al. (44)	Crizotonib vs. alectinib	Retrospective cohort	164	—	Time-to-CNS progression longer in alectinib vs. crizotinib (HR, 0.19; 95% CI: 0.07–0.53; *p* = 0.0004).
	Zhao et al. (45)	Icotinib vs. chemo	Retrospective cohort	396	Lower	IMD incidence at 24 months lower for icotinib (10.2%) vs. chemotherapy (32.1%). Hazard ratio for IMD in chemotherapy vs. icotinib 3.32 (95% CI 1.89–5.82; *p* < 0.001).
RCC	Verma et al. (46)	TKI vs. no TKI	Retrospective cohort	338	Lower	IMD incidence lower in TKI vs. no TKI (HR, 0.39; 95% CI 0.21–0.73; *p* = 0.003).
	Dudek et al. (33)	TKI vs. no TKI	Retrospective cohort	92	Lower	IMD incidence lower in TKI vs. no TKI (per month incidence rate ratio 1.568; 95% CI 1.06–2.33).
	Massard et al. (47)	Sorafenib vs. placebo	Retrospective cohort	139	Lower	IMD incidence lower in sorafenib (3%) vs. placebo (12%, *p* < 0.05).
	Vanhuyse et al. (48)	Antiangiogenic^**^ vs. other therapy	Retrospective cohort	199	—	IMD incidence in targeted therapy group (15.7%) lower than non-targeted therapy group (18.2%). However, targeted therapy was not associated with a lower cumulative rate of brain metastases (HR, 0.58; 95% CI 0.26–1.30; *p* = 0.18).
HCC	Shao et al. (49)	Antiangiogenic therapy ^***^	Retrospective cohort	158	Higher	IMD incidence 7% in patients receiving antiangiogenic targeted therapies vs. 0.2–2.2% in historical comparators. Median time-to-IMD 9.6 months.

*- Incidence trends marked with a dash if study reports 1) insignificant results, 2) only comparison between multiple targeted therapies, or 3) no control*.

* *Both Viani et al. and Bria et al. report on the same datasets*.

** *Antiangiogenic therapies in Vanhuyse et al. study = sorafenib, sunitinib, bevacizumab, temsirolimus, or everolimus*.

*** *Antiangiogenic therapies in Shao et al. study = sorafenib, sorafenib plus tegafur/uracil, sunitinib, bevacizumab plus capecitabine, bevacizumab plus erlotinib, or thalidomide plus tegafur/uracil*.

*(cs)HR, (cause-specific) hazard ratio; RR, relative risk; RCT, randomized controlled trial; NSCLC, non-small cell lung cancer; RCC, renal cell carcinoma; HCC, hepatocellular carcinoma; TKI, tyrosine kinase inhibitor (sorafenib or sunitinib); EGFR inhibitor, gefitinib or erlotinib; BRAF/MEK inhibitor, BRAF, vemurafenib or dabrafenib; MEK, cobimetinib or trametinib*.

The current literature is also mixed on whether targeted therapies increase, decrease, or have any impact on the incidence of IMD incidence. Many studies report insignificant differences in IMD incidence between patients receiving a targeted therapy vs. a conventional chemotherapy (14, 34, 35, 39, 48). In breast cancer, most studies indicate that targeted therapy is associated with increase in IMD incidence (29, 35, 38). One study reports a prolonged median time-to-IMD in patients receiving targeted therapy vs. other therapies, supporting the “sanctuary” hypothesis that prolonged survival due to systemic disease control increases IMD risk (35). In RCC, targeted therapy is associated with a decrease in IMD incidence (33, 46, 47). In non-small cell lung cancer, some studies report an increase in IMD incidence with use of a targeted therapy, while others report an associated decrease (28, 31, 42, 43). One hypothesis to explain these differences between primary disease types is that the targeted therapies studied in breast cancer, such as the 140kDa+ monoclonal antibodies trastuzumab and pertuzumab, are less BBB-penetrant than available targeted therapies in RCC, such as the small molecule kinase inhibitors sunitinib and sorafenib, while there is a range of BBB-penetrability among the therapies used in non-small cell lung cancer. However, the arrival of novel BBB-penetrant agents may be anticipated to disrupt these trends.

## Future Directions

The questions of IMD incidence and survival are relevant today because the frequency of IMD is rising, while prognosis remains poor (3). As improvements are made in the early detection of IMD and the management of systemic disease, more clinicians will counsel patients on the risk and management of IMD. Additionally, the use of targeted therapies is expected to increase as the management of both primary systemic disease and IMD moves toward precision methods, raising the question of the impact of targeted therapies on IMD incidence and survival (11). Formal appraisal to date has found insufficient evidence for the use of targeted therapies in the treatment of IMD, and the question of IMD incidence following targeted therapy remains debated (19).

Future studies may address these gaps from multiple approaches. Trials of targeted therapies have historically excluded patients with baseline IMD, but more recent studies have done so, beginning the process of clarifying the role of targeted therapy in the management of this disease. Prospective collection of data on intracranial outcomes in patients treated with a targeted therapy will elucidate the risk of IMD and provide insight on the role of targeted therapy in treating IMD. Future retrospective studies interested in the question of IMD incidence may examine larger populations to more finely control for covariates like cancer mutation status, or compare the effects of targeted therapies across primary disease types. Meta-analyses will benefit from broader reporting of IMD incidence stratified by status of baseline CNS disease, and database studies will allow observation of longer-term outcomes across institutions as survival with IMD improves.

While the 2019 guidelines from the Congress of Neurological Surgeons do not make recommendations on the use of targeted therapy in the management of IMD, they note in their evidence review that therapies and studies since 2015 were not considered. Yet, targeted therapies in the field of IMD have undergone explosive development since that time, with new approvals in breast cancer, non-small cell lung cancer, melanoma, and RCC. New data will clarify the role of targeted therapy in the initial treatment of IMD, and clinicians will be required to make complex management decisions considering treatment sequencing, multimodal strategies with radiation and surgery, and weighing survival and quality of life for their patients with IMD. As survivorship in primary disease improves, more physicians may expect to discuss IMD risk with patients receiving targeted therapy, or to consider the implementation of focused surveillance imaging. Targeted therapy may replace the frontline modalities in the management of IMD, or it may occupy a prophylactic role for patients with primary disease. More immediately, targeted therapy may fill adjuvant or neoadjuvant roles alongside the current standard IMD treatments, and may vary between primary disease types.

## Conclusion

Targeted therapies are emerging onto a dynamic treatment landscape for IMD, and future work will elucidate their place among current standards. Present data are few on IMD incidence among patients receiving targeted therapies for primary cancers, often limited to studies with single arms or small sample sizes. Future studies will stratify IMD incidence according to the BBB penetrance of targeted therapies in order to clarify the role of targeted therapies in preventing—or facilitating—the development of IMD. There is also a need for larger studies with higher power to elucidate the impact of targeted therapy on both incidence and survival in IMD. As more novel agents are developed, and the management of systemic disease improves, the treatment landscape for IMD may be expected to change, and physicians may anticipate considering IMD risk as they create management plans and counsel patients.

## Author Contributions

AE and SD wrote and revised the manuscript. AE was responsible for review of the literature and drawing the table and figures. SD designed the article.

### Conflict of Interest Statement

The authors declare that the research was conducted in the absence of any commercial or financial relationships that could be construed as a potential conflict of interest.
