# Supplementary material for: The Impact of Targeted Therapy on Intracranial Metastatic Disease Incidence and Survival
Source: Front Oncol. 2019 Aug 23;9:797. doi: 10.3389/fonc.2019.00797 (PMC6716495; doi:10.3389/fonc.2019.00797)
Supplement: Supplementary file 1 [file Table_1.DOCX]

**Appendix 1.** Database search query for IMD incidence with targeted therapy.

| Operator | Terms | Field |
| --- | --- | --- |
|  | develop* OR incid* OR preven* | Title |
| AND | brain OR intracranial OR cerebral | Title |
| AND | metasta* | Title |
| AND | target* or therapy OR trastuzumab OR ramucirumab OR atezolizumab OR nivolumab OR durvalumab OR avelumab OR pembrolizumab OR bevacizumab OR tamoxifen OR toremifene OR fulvestrant OR anastrozole OR exemestane OR lapatinib OR letrozole OR pertuzumab OR ado-trastuzumab OR T-DM1 OR palbociclib OR ribociclib OR neratinib OR abemaciclib OR olaparib OR cetuximab OR panitumumab OR aflibercept OR regorafenib OR ipilimumab OR imatinib OR lanreotide OR avelumab OR sunitinib OR denosumab OR sorafenib OR pazopanib OR temsirolimus OR axitinib OR cabozantinib OR lenvatinib OR tretinoin OR dasatinib OR nilotinib OR bosutinib OR alemtuzumab OR ofatumumab OR obinutuzumab OR ibrutinib OR idelalisib OR blinatumomab OR venetoclax OR ponatinib OR enasidenib OR inotuzumab OR tisagenlecleucel OR gemtuzumab OR ivosidenib OR duvelisib OR moxetumomab OR glasdegib OR gilteritinib OR crizotinib OR erlotinib OR gefitinib OR afatinib OR ceritinib OR ramucirumab OR osimertinib OR necitumumab OR alectinib OR brigatinib OR trametinib OR dabrafenib OR durvalumab OR dacomitinib OR lorlatinib OR ibritumomab OR brentuximab OR vorinostat OR romidepsin OR bexarotene OR bortezomib OR pralatrexate OR siltuximab OR belinostat OR copanlisib OR acalabrutinib OR mogamulizumab OR carfilzomib OR panobinostat OR daratumumab OR ixazomib OR elotuzumab OR ruxolitinib OR dinutuximab OR rucaparib OR niraparib OR cabazitaxel OR enzalutamide OR abiraterone OR apalutamide OR vismodegib OR sonidegib OR trametinib OR cobimetinib OR alitretinoin OR avelumab OR encorafenib OR binimetinib OR cemiplimab OR olaratumab OR larotrectinib OR vandetanib | All fields |

Search conducted on EMBASE and MEDLINE databases on June 7^th^, 2019.
